# Application value of multi-parameter magnetic resonance image-transrectal ultrasound cognitive fusion in prostate biopsy

**DOI:** 10.1515/med-2024-1026

**Published:** 2024-09-13

**Authors:** Haichuan Yuan, Min Huang, Tao Liu, Wu Song, Chengpeng Luo

**Affiliations:** Department of Urology, Renhe Hospital, Shanghai, 200431, People Republic of China; Preventive Health Care Section, Gaojing Town Community Health Service Center, Shanghai, 200435, People Republic of China; Department of Urology, Youyi Street Community Health Service Center, Shanghai, 201999, People Republic of China; Department of Urology, Renhe Hospital, Baoshan District, 1999 West Changjiang Road, Shanghai, 200431, People Republic of China

**Keywords:** three-dimensional reconstruction, prostate cancer, cognitive fusion, targeted biopsy, systematic biopsy

## Abstract

**Objective:**

To investigate the effect of three-dimensional (3D) reconstruction-assisted cognitive fusion in targeted prostate biopsy.

**Results:**

There was no significant difference in the detection rate of prostate cancer (PCa) between targeted biopsy and systematic biopsy, and there was significant difference in the detection rate of clinically significant prostate cancer (csPCa) between targeted biopsy and systematic biopsy. In the low prostate total specific antigen (tPSA) group, there was no statistically significant difference in the detection rate of prostate cancer between the two biopsy modalities. However, compared with systematic puncture, targeted puncture had a higher detection rate for csPCa and a lower detection rate for clinically insignificant prostate cancer (ciPCa), and the difference was statistically significant. In the high tPSA group, there was no significant difference in the detection rate of PCa, csPCa, and ciPCa between the two biopsy types. Single needle positive rate of targeted puncture (29.77%) was significantly higher than that of systematic puncture (10.28%).

**Conclusions:**

The detection rate of csPCa in 3D reconstruction-assisted cognitive fusion targeted prostate biopsy is better than that of 12-needle systematic biopsy, which markedly improved the positive rate of prostate biopsy.

## 1 Introduction

Prostate cancer (PCa) is an epithelial malignant tumor occurring in the prostate [1]. PCa is a common malignant tumor in middle-aged and elderly men, ranking second in male malignant tumor incidence and mortality, second only to lung cancer, and its incidence has been increasing year by year in recent years [2]. Presently, although the incidence and mortality of PCa in European and American countries are higher than that in China, investigation and research showed that the proportion of newly diagnosed patients with middle or advanced PCa is much lower than that in western countries, and the resulting missed diagnosis or false negative is also the reason for the poor prognosis of patients [3,4]. In terms of the number of puncture needles, the researchers increased the sampling of other prostate tissues on the basis of the original 6 needles, resulting in a layout scheme of 12 needles or even more than 20 needles of saturated puncture. Although these layouts significantly improved the detection rate of clinically significant prostate cancer (csPCa), the higher number of needles also resulted in an increased risk of complications. Studies have shown that fewer systematic biopsy cores can lead to lower csPCa detection rates and changes in focal treatment plans, especially when performing MRI-targeted biopsies [5]. Transrectal ultrasound (TRUS)-guided systematic prostate biopsy is the preferred method for the diagnosis of PCa [6]. However, due to the multifocal and heterogeneous characteristics of early cancer lesions, false negative results may still appear even if systematic puncture or saturated puncture biopsy is adopted. Therefore, 22–47% of PCa are missed in the first puncture [7]. For this reason, improving the diagnosis rate of early PCa and the prognosis of PCa patients has become a major problem that medical workers are facing urgently.

With the development of imaging technology, multi-parameter magnetic resonance imaging (mpMRI) has provided a more effective diagnostic method for PCa, especially in the early diagnosis of csPCa of clinical significance, which can provide more accurate spatial localization of the tumor on the basis of qualitative diagnosis [8,9]. Targeted puncture technology is a fusion technology combining mpMRI imaging and TRUS real-time guidance. Compared with traditional biopsy technology, targeted puncture has higher sensitivity and diagnostic accuracy for PCa [10,11]. At present, the rapid development of computer three-dimensional (3D) reconstruction technology has rapidly penetrated into many industries including the medical industry [12,13]. The increasing sophistication of 3D medical imaging models, efficient 3D computer rendering, multi-dimensional volume-image data modeling, and image-guided navigation are significantly increasing the ability for minimally invasive diagnosis and treatment [14].

This study evaluated the diagnosis of PCa in patients with PI-RADS score ≥3 by mpMRI image reconstruction-TRUS cognitive fusion prostate targeted biopsy and systematic prostate biopsy, aiming to explore the clinical value of targeted biopsy in detecting PCa and csPCa, and provide valuable data for the promotion of this examination method in the diagnosis of PCa.

## 2 Materials and methods

### 2.1 Subject inclusion

The clinical data of 103 patients with TRUS-guided perineal prostatic puncture biopsy admitted to the Department of Urology of Renhe Hospital, Baoshan District from January 2019 to December 2021 were retrospectively analyzed, and the enrolled patients all met the following conditions: (1) preoperative serum total prostate specific antigen (tPSA) >4 ng/mL, and abnormal nodule signal was found by mpMRI before puncture (at least one suspicious lesion); (2) PI-RADS score ≥3; and (3) no history of prostate puncture, all patients underwent prostate puncture biopsy for the first time. Exclusion criteria included: (1) there are contraindications for puncture (coagulation dysfunction, serious cardiovascular, and cerebrovascular diseases); (2) there are factors that affect the results of tPSA examination; and (3) MRI cannot be performed.

### 2.2 Imaging examinations

All patients had completed mpMRI before puncture. Prostate MRI scanner (Skyra 3.0T MR, SIEMENS, Germany) was used for examination, and the scanning sequence included T1WI, T2WI, DWI, ADC, DCE, etc. The DCE-MRI scan consisted of seven phases (one phase of plain scan and six phases of scan after injection of contrast agent). The scan included the prostate area and the seminal vesicle gland. T2WI was the dominant sequence in the score of transitional zone lesions, and DWI and ADC were the dominant reference sequence in the score of peripheral zone lesions. Two urological radiologists with more than 10 years of clinical experience reviewed the MRI images, identified the suspected lesions through negotiation, and scored the suspected areas according to PI-RADS V2. These two radiologists did not know information about the patient, including previous prostate biopsy and PSA levels. The doctor performed the puncture using a transrectal biplanar ultrasound, perineal positioning template, and a 22 mm 18G disposable automatic biopsy gun. Abnormal prostate nodule signals were found on imaging examination, and abnormal signal areas were marked.

### 2.3 3D image reconstruction and design of puncture path

**Figure 1:**
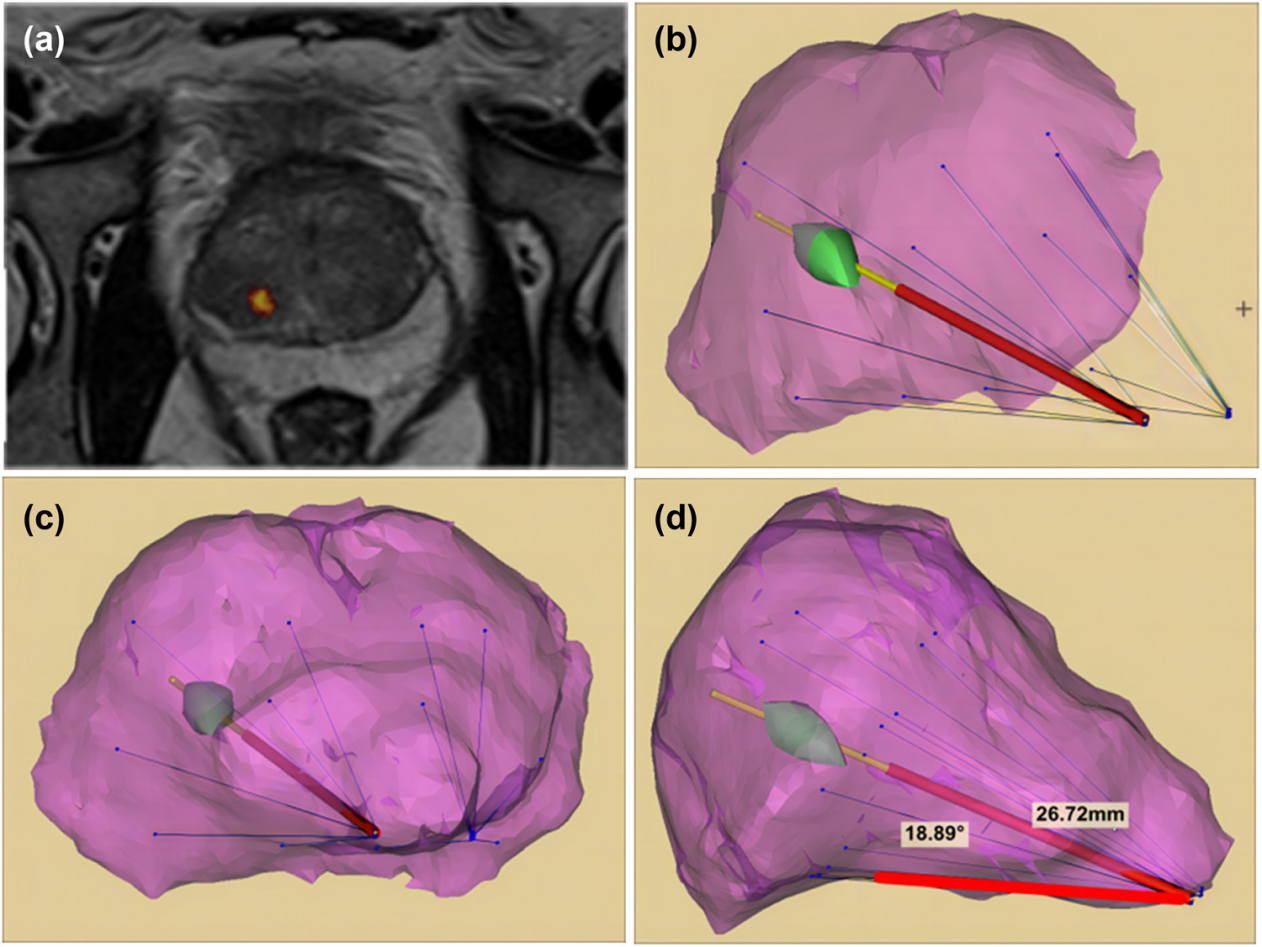
3D reconstruction of prostate images and puncture path design. (a) Both T2 weighted mpMRI and DCE indicated suspicious abnormal signals in the right posterior peripheral zone of the prostate, and the two images were fused to mark the suspicious lesion location. (b and c) 3D reconstruction of image data is carried out by Mimics18.0 medical image processing software. Purple represents the simulated prostate image, green represents the simulated suspicious lesion image, and blue line represents the simulated puncture path of 12-needle system. (d) Sagittal plane was used to determine the targeted puncture path. The insertion distance from the prostate capsule was about 26.72 mm, and the angle from the ultrasound probe plane was about 18.89°.

The MRI image data of the prostate in DI-COM format was imported into the Mimics18.0 medical image processing software, and 3D image reconstruction was carried out for the prostate and suspicious nodules’ images. The targeted puncture path and needle depth were designed according to the size and location of suspicious lesions in the 3D image, and 1–2 needles were designed for each suspicious lesion (Figure 1). The difficulty of this operation lies in the recognition of suspicious lesions on MRI and the understanding of ultrasonic scanning space, which requires the operator to have a certain basis of specialist imaging diagnosis and local anatomy.

### 2.4 Prostatic puncture method

All patients underwent general anesthesia, perineal puncture biopsy, and were performed by the same urologist. The puncture was performed according to the design scheme. The patient took the lithotomy position, routinely disinfected the puncture site, and fixed the puncture guide on the body surface. Subsequently, targeted puncture path designed by 3D reconstruction was performed on suspicious lesions found by mpMRI under cognitive fusion. (The vertical distance between the suspected lesion and the urethra was determined through the coronal plane and cross-section of mpMRI. The cross-section of the suspected lesion was found using the puncture rack stepper. The coordinates of the central point of the lesion were determined according to the *X*-axis and *Y*-axis of ultrasound.) The Bard 18G disposable puncture gun was applied to puncture each target with 2–3 needles through the perineum. After the targeted puncture was completed, the patient underwent ultrasound-guided perineal 12-needle prostatic system puncture immediately. After finding the maximum cross-section of the prostate under ultrasound, five needles were biopsied on the left and right side of the prostatic projection area as close to the edge and the posterior side as possible, and one needle was punctured on both sides of the internal gland. Antibiotics were routinely applied after surgery.

### 2.5 Pathological grouping

All specimens were labeled and fixed with 10% formaldehyde solution and sent for pathological examination. Those who were finally diagnosed with PCa by pathology were classified by Gleason score, and those with Gleason score lower than or equal to 6 were classified as clinically insignificant prostate cancer (ciPCa), and those with Gleason score higher than or equal to 7 were csPCa, and radical prostatectomy was performed for these patients.

### 2.6 Statistical analysis

The pathological positive data of systematic puncture and targeted puncture were recorded into SPSS 20.0 statistical software, and the comparison of categorical variables between groups was analyzed by Pearson *χ* ^2^. The detection rates of systematic puncture and 3D reconstruction cognitive fusion targeted puncture for PCa, csPCa, and ciPCa were evaluated. *P* < 0.05 was considered statistically significant.


**Informed consent:** All subjects have given informed consent.
**Ethical approval:** The procedure followed in this study has been approved by the Ethics Committee of this hospital and conforms to the requirements of the Helsinki Declaration of the World Medical Association.

## 3 Results

### 3.1 General information of all subjects

As shown in Table 1, patients who were to undergo prostate biopsy ranged in age from 55 to 88 years, with an average age of (71.52 ± 8.29) years, and tPSA levels ranged from 4.02 to 670.29 ng/mL, with an average value of (57.31 ± 113.88) ng/mL. Besides, the median time to biopsy after mpMRI was 13 (3, 34) days. There were 10 (9.71%) patients with positive digital rectal examination, 52 (50.49%) patients with PI-RADS score of 3, 34 (33.01%) patients with score 4, and 17 (16.50%) patients with score 5.

**Table 1:** Basic information of patients

Characteristics	All patients (*n* = 103)
Age (years)	71.52 ± 8.29
tPSA (ng/mL)	57.31 ± 113.88
DRE-positive (*n*, %)	10 (9.71%)
PI-RADS 3 (*n*, %)	52 (50.49%)
PI-RADS 4 (*n*, %)	34 (33.01%)
PI-RADS 5 (*n*, %)	17 (16.50%)
Time from mpMRI to biopsy	13 (3, 34)

Abbreviations: tPSA, total prostate specific antigen; DRE, digital rectal examination; PI-RADS, prostate imaging reporting and data system. The measurement data are expressed as mean ± standard deviation (SD) and median (range), and the counting data are expressed as *n* (%).

### 3.2 Comparison of detection rates of PCa by different puncture types

As shown in Table 2, there were 65 PCa cases in the systematic puncture group and 67 PCa cases in the targeted puncture group, and the difference was not statistically significant (*P* > 0.05). Among PCa diagnosed by puncture method, 41 csPCa cases were determined in the systematic puncture group, while the number of csPCa determined in the targeted puncture group (60 cases) was significantly higher than that in the systematic puncture group (*P* < 0.05). In addition, the number of ciPCa diagnosed in the targeted puncture group (7 cases) was significantly lower than that in the systematic puncture group (24 cases) (*P* < 0.05).

**Table 2:** Comparison of detection rates of PCa by different puncture types

Cancer types	Targeted puncture	Systematic puncture	*χ* ^*2*^	*P*
PCa (*n*, %)	67 (65.05%)	65 (63.11%)	0.084	0.771
csPCa (*n*, %)	60 (58.25%)	41 (39.81%)	7.012	0.012
ciPCa (*n*, %)	7 (6.80%)	24 (23.30%)	10.974	0.001

Abbreviations: PCa: prostatic cancer; csPCa: clinically significant prostatic cancer; ciPCa: clinically insignificant prostate cancer. Notes: *P* < 0.05 means significant difference.

### 3.3 Comparison of PCa detection rates in different tPSA groups with different puncture types

All subjects were divided into two groups according to preoperative tPSA: 40 cases in tPSA 4–10 ng/mL group (low tPSA group) and 63 cases in tPSA >10 ng/mL group (high tPSA group). In the low tPSA group, 18 and 17 cases of PCa, 15 and 6 cases of csPCa, and 3 and 11 cases of ciPCa were detected by targeted puncture and systematic puncture, respectively. There was no significant difference between the two puncture methods in the detection of PCa (*P* > 0.05), but the significant difference was observed in the detection rate of csPCa and ciPCa (*P* < 0.05). Meanwhile, in the high tPSA group, 49 and 48 cases of PCa, 44 and 36 cases of csPCa, and 5 and 12 cases of ciPCa were detected by targeted puncture and systematic puncture, respectively. There was no significant difference in the detection rate of PCa, csPCa, and ciPCa between the two puncture methods (*P* > 0.05, Table 3).

**Table 3:** Comparison of PCa detection rates in different preoperative tPSA groups with different puncture types

Group	Cancer type	Targeted puncture	Systematic puncture	*χ* ^*2*^	*P*
Low tPSA (*n* = 40)	PCa	18 (45.00%)	17 (42.50%)	0.051	0.829
	csPCa	15 (37.50%)	6 (15.00%)	5.230	0.041
	ciPCa	3 (7.50%)	11 (27.50%)	5.541	0.037
High tPSA (*n* = 63)	PCa	49 (77.78%)	48 (76.19%)	0.045	0.859
	csPCa	44 (69.84%)	36 (57.14%)	2.191	0.195
	ciPCa	5 (7.94%)	12 (19.05%)	3.332	0.116

Abbreviations: tPSA: total prostate specific antigen; PCa: prostatic cancer; csPCa: clinically significant prostatic cancer; ciPCa: clinically insignificant prostate cancer. *P* < 0.05 means significant difference.

### 3.4 Comparison of single needle positive rate between targeted puncture and systematic puncture

As shown in Table 4, the total number of needles for targeted puncture was 299, of which 89 were positive, and the positive rate of single needle was 29.77%. However, the total number of needles for systematic puncture was 1,236, of which 127 were positive, and the positive rate of single needle was 10.28%. The positive rate of single needle in the targeted puncture group was significantly higher than that in the systematic puncture group (*P* < 0.05), indicating that targeted puncture could obtain a higher positive rate by using fewer puncture needles. Furthermore, according to the above information, the average number of biopsy cores for each patient was calculated, which was 2.90 ± 0.30 in the targeted puncture group and 12 in the systematic puncture group, and the difference was significant (*P* < 0.001). The mean number of positive cores in targeted and systematic puncture patients was 1.33 ± 0.47 and 1.95 ± 0.83 (*P* < 0.001). The mean positive rates of patients with targeted and systematic puncture were 0.48 ± 0.19 and 0.16 ± 0.07 (*P* < 0.001). The positive rate of targeted biopsy was higher than that of systematic biopsy.

**Table 4:** Comparison of single needle positive rate of two puncture methods

	Targeted puncture	Systematic puncture	*P*
Total number of puncture needles	299	1,236	—
Total number of positive needles	89 (29.77%)	127 (10.28%)	0.006
Average number of biopsy cores per patient	2.90 ± 0.30	12.00 ± 0.00	<0.001
Average number of positive cores per patient	1.33 ± 0.47	1.95 ± 0.83	<0.001
Average positive rates per patient	0.48 ± 0.19	0.16 ± 0.07	<0.001

Note: *P* < 0.05 was considered as significant difference.

## 4 Discussion

TRUS-guided prostate puncture biopsy has been the first choice for the diagnosis of PCa due to its advantages of low equipment requirements and simple operation [15]. However, among the patients who adopt this method, there are still some people who have not been detected with PCa. The reason for this result is that there is a certain sampling blind area in anterior zone and apex of the prostate and the vicinity of urethra, and insufficient sampling in this area may easily lead to missed diagnosis of PCa [16]. The ideal PCa detection method should be minimally invasive, few complications, and be able to distinguish between csPCa and ciPCa as accurately as possible, so that the majority of patients with csPCa can benefit from further treatment and minimize overtreatment of patients with ciPCa [17,18]. However, in terms of current clinical research and medical technology, more clinical trials and studies are still needed to provide more evidence on the best prostate biopsy strategy.

In this study, in order to obtain the 3D virtual image model of the patient’s prostate and suspicious lesions, the two-dimensional (2D) image of the patient’s mpMRI was analyzed and reconstructed by using 3D reconstruction and visualization software. On this basis, according to the shape, size, and spatial position of the lesion and gland, we can accurately design the individualized needle insertion angle, depth, and puncture path. In the present study, it was found that the detection rate of csPCa by 3D reconstruction-assisted cognitive fusion targeted puncture was 58.25%, which was significantly higher than that of systematic puncture (39.81%). Furthermore, according to the tPSA value, the detection rate of csPCa by targeted puncture was also significantly higher than that by systematic puncture in the group of 4 ng/mL < tPSA ≤ 10 ng/mL. However, there was no significant difference in the detection rates of PCa, ciPCa, and csPCa between targeted puncture and systematic puncture in tPSA > 10 ng/mL group. We speculate that the reasons for this result may be attributed to two aspects. First, high levels of tPSA are associated with greater lesion degree, and theoretically, with increasing tPSA levels, the likelihood of developing major cancers is increasing, indicating that biopsy puncture is sufficient to detect major cancers, whether it is targeted puncture or systematic puncture. Second, the sample size of this study is small and retrospective, which may cause selection bias in the process of sample inclusion.

MpMRI is an imaging technology with high resolution, high sensitivity, and high specificity, which can accurately detect the position of PCa and stratify the risk, provide accurate image guidance for targeted prostate biopsy, and improve the detection rate of csPCa in imaging [19,20]. At the same time, through the comprehensive evaluation of DCE, DWI, and T2WI images, the focus can be located accurately in space [21]. A series of MRI-assisted prostate puncture applications, such as mpMRI-TRUS cognitive fusion targeted puncture, mpMRI-TRUS software fusion targeted puncture, and mpMRI-directly guided targeted puncture and a series of other MRI-assisted prostate puncture make up for the defects of systematic puncture random sampling in the past, and can effectively improve the detection rate of PCa, especially csPCa [22,23]. For a long time, the debate about targeted biopsy and systematic biopsy is not uncommon. Lee et al. reported in a retrospective study that MRI-US targeted biopsy and systematic biopsy based on the transperineal robot-assisted prostate biopsy platform showed no statistical difference in the overall detection rates of PCa and csPCa, but targeted biopsy had significantly higher sampling efficiency than systematic biopsy [11]. In a large cohort study, Brown et al. found an 18% increase in the detection rate of PCa with mpMRI-TRUS cognitive fusion targeted puncture compared to TRUS-guided prostate puncture [24]. At present, the commonly used detection method in clinical practice is targeted combined systematic biopsy. Studies have shown that the positive rate of combined biopsy and the detection rate of PCa are significantly higher than that of single targeted biopsy and systematic biopsy [25]. However, a possible problem with combination biopsies is the possibility of resampling in systematic biopsies. It has been reported that repeated sampling of the MRI area of interest by systematic puncture leads to a decrease in the detection rate of csPCa by systematic biopsy, while an increase in the detection rate of csPCa by targeted puncture [26]. Other studies have found that perifocal biopsies can improve the detection of csPCa and reduce overdiagnosis of ciPCa. In a retrospective analysis, Lee et al. found that MRI-based focal saturated biopsy strategies can improve the detection rate of csPCa and reduce the grading at prostatectomy [27].

Traditional TRUS-guided prostate puncture includes transrectal puncture and perineal puncture [28]. Since transrectal puncture often leads to postoperative bleeding and infection, perineal puncture has gradually replaced transrectal puncture in current medical means. It is worth noting that the puncture method mentioned above requires a dedicated image fusion and puncture platform and is still based on the cognitive basis of 2D images, which still has certain limitations. 3D reconstruction of medical image refers to the process of establishing 3D visual models of organs or tumors from the slices of organs or tumors by 3D reconstruction technology [29]. 3D reconstruction technology enables doctors to evaluate the target tissue more comprehensively, which is conducive to the planning and arrangement of treatment programs. The 3D reconstruction technology based on mpMRI-TRUS cognitive fusion targeted puncture can transform the organ structure into a digital model for observation, measurement, and even reverse analysis after 3D reconstruction [30]. 3D reconstruction can accurately model and locate the mpMRI diagnostic information. In this study, 3D reconstruction assisted cognitive fusion avoided missed diagnosis of csPCa in 19 cases of prostate targeted puncture, greatly improving the positive rate of biopsy. At the same time, a total of 299 needles were pierced by targeted puncture, with a single needle positive rate of 29.77%, while a total of 1,236 needles were pierced by systematic puncture, with a single needle positive rate of 10.28%. There were significant differences in the single needle positive rates of the two puncture methods. A higher positive rate of a single needle can be achieved by using a smaller number of puncture needles using targeted puncture techniques. In the future, with the standardization of mpMRI image analysis and the accumulation of doctors’ experience, targeted puncture technology will be gradually applied to the medical process.

The limitations of this study are as follows. On the one hand, the sample size of this study is small, and it is a retrospective single-center study, which is less scientific than the results of multi-center and large-sample prospective studies. On the other hand, the issue of operator bias needs to be addressed, especially when performed by the same urologist. Since the systematic biopsy puncture was conducted after the targeted biopsy puncture, the operator’s understanding of the target location may influence the puncture location of the systematic biopsy puncture needle. Therefore, in future studies, multi-center, large-sample size prospective studies should be considered to evaluate the detection rates of PCa and csPCa by targeted puncture and systematic puncture. At the same time, in order to avoid the occurrence of operator bias, according to previous studies, 12-needle system puncture should be performed first, and then targeted puncture [31].

In conclusion, we used computer 3D reconstruction technology to plan and simulate the puncture process in advance, and guided doctors to carry out accurate targeted operation through TRUS dynamic image cognitive fusion during puncture. The puncture positive rate of csPCa (especially tPSA located in gray area) was significantly higher than that of system puncture. The development of new technology and the integration of multiple disciplines is the necessary force for the development of diagnosis and treatment in the medical field. Computer 3D reconstruction technology will be more widely used in the early diagnosis of CSPcas with tPSA located in gray area. In the future, the popularization and development of new technologies will benefit more patients.
